# Metabolomic mechanisms of gypenoside against liver fibrosis in rats: An integrative analysis of proteomics and metabolomics data

**DOI:** 10.1371/journal.pone.0173598

**Published:** 2017-03-14

**Authors:** Ya-Nan Song, Shu Dong, Bin Wei, Ping Liu, Yong-Yu Zhang, Shi-Bing Su

**Affiliations:** 1 Research Center for Traditional Chinese Medicine Complexity System, Shanghai University of Traditional Chinese Medicine, Shanghai, China; 2 E-institutes of Traditional Chinese Internal Medicine, Shanghai Municipal Education Commission, Shanghai University of Traditional Chinese Medicine, Shanghai, China; 3 Research Center for Traditional Chinese Medicine and System Biology, Shanghai University of Traditional Chinese Medicine, Shanghai, China; IDIBAPS Biomedical Research Institute, SPAIN

## Abstract

**Aims:**

To investigate mechanisms and altered pathways of gypenoside against carbon tetrachloride (CCl_4_)-induced liver fibrosis based on integrative analysis of proteomics and metabolomics data.

**Methods:**

CCl_4_-induced liver fibrosis rats were administrated gypenoside. The anti-fibrosis effects were evaluated by histomorphology and liver hydroxyproline (Hyp) content. Protein profiling and metabolite profiling of rats liver tissues were examined by isobaric tags for relative and absolute quantitation (iTRAQ) approach and gas chromatography-mass spectrometer (GC-MS) technology. Altered pathways and pivotal proteins and metabolites were searched by integrative analysis of proteomics and metabolomics data. The levels of some key proteins in altered pathways were determined by western blot.

**Results:**

Histopathological changes and Hyp content in gypenoside group had significant improvements (*P*<0.05). Compared to liver fibrosis model group, we found 301 up-regulated and 296 down-regulated proteins, and 9 up-regulated and 8 down-regulated metabolites in gypenoside group. According to integrative analysis, some important pathways were found, including glycolysis or gluconeogenesis, fructose and mannose metabolism, glycine, serine and threonine metabolism, lysine degradation, arginine and proline metabolism, glutathione metabolism, and sulfur metabolism. Furthermore, the levels of ALDH1B1, ALDH2 and ALDH7A1 were found increased and restored to normal levels after gypenoside treated (*P*<0.05).

**Conclusions:**

Gypenoside inhibited CCl_4_-induced liver fibrosis, which may be involved in the alteration of glycolysis metabolism and the protection against the damage of aldehydes and lipid peroxidation by up-regulating ALDH.

## Introduction

Liver fibrosis is a common consequence of chronic liver injury resulting from multiple etiology[1]. Long-term unresolved liver fibrosis can lead to liver cirrhosis, and even hepatocellular carcinoma[2]. Therefore, the development of liver fibrosis is a key step to determine the clinical outcome of chronic liver diseases. Control or resolution of liver fibrosis is an important problem in order to avoid the development of terminal illness.

Gypenoside, a saponin extract derived from *Gynostemma pentaphyllum* (Thunb.) Makino., a traditional Chinese medicine, has been reported to have hepatoprotective effect via numerous bioactivities, such as anti-fibrotic activity [3], anti-oxidative and anti-apoptotic activity [4]. Mostly previous studies focused on the pharmacology and mechanisms of molecules for gypenoside protecting against liver injury. However, physiological alterations resulting from chemical substances don’t affect only a few functionally important biomolecules. Instead, physiological condition is usually affected in the whole molecular network and multiple pathways[5]. Thus, it is conceivable that the identification of deregulated pathways is a fruitful discovery approach, as a starting point for later analysis of molecular mechanisms.

Omics techniques are powerful tools for clarifying pathogenesis and identifying biomarkers for diseases[6, 7]. Proteome reveals all proteins and peptides, and metabolome reflects all endogenous metabolites. Thus, integrative analysis of proteomics and metabolomics can conduct a more detailed evaluation of physiological status, especially from the global and overall perspective. Lots of researchers have studied the altered network and pathways of pathological or medical status using proteomics and metabolomics[8, 9].

In this study, we analyzed liver tissues of carbon tetrachloride (CCl_4_)-induced liver fibrosis in rats on a global scale, in terms of the relative expression of proteins based on isobaric tags for relative and absolute quantitation (iTRAQ) approach and relative metabolite abundances based on gas chromatography-mass spectrometer (GC-MS) technology, aiming to explore the metabolomic mechanisms of gypenoside against liver fibrosis in rats underlying the global changes of proteome and metabolome.

## Methods

### Animal experiments

Male Wistar rats (180–200 g, n = 30) were housed in standard animal conditions with 12 h/12 h light/dark cycle and constant temperature. All of them were randomly separated into two groups, control group (n = 10) and CCl_4_-treated group (n = 20). In CCl_4_-treated group, a 1:1 solution of CCl_4_ and olive oil (2ml/kg) was administered intraperitoneally twice a week for 9 weeks, and control group received equal quantities of physiological saline. At the end of the 6th week, CCl_4_-treated rats were randomly assigned to two groups, model group and gypenoside-treated group (10 rats in each group). With continued 3-week CCl_4_ administration, the rats received a daily treatment with water or 200 mg/kg gypenoside intragastrically. At the end of the 9th week, all rats were sacrificed and liver tissue samples were collected for further analysis. The study protocol was approved by the Animal Ethics Committee of Shanghai University of Traditional Chinese Medicine.

### Histomorphology and hepatic hydroxyproline content

4% paraformaldehyde-fixed liver tissues were processed as following: dehydrated, embedded, sectioned, and finally stained with Hematoxylin and Eosin (H&E) and Sirius red. Specimens were observed for histopathological changes.

Liver tissue specimen (100 mg) was performed for hydroxyproline (Hyp) determination following a modified method by Jamall [10]. Hyp content represented an indirect value of tissue collagen content, and was expressed as μg/g wet weight (μg/g).

### Proteomics

Procedures of the iTRAQ-mass spectrometry are described in detail in [11, 12]. Briefly, a double duplex iTRAQ experiment was performed using the four-plex iTRAQ reagent (iTRAQ Reagents Multiplex Kit, Applied Biosystems, Foster City, CA). Proteins from duplicate sets were isolated and equal amounts of proteins were used for labeling by iTRAQ reagents followed manufacturer’s instructions (Applied Biosystems). After labeling, all four samples were mixed, and separated by strong cation exchange (SCX) and reverse phase liquid chromatography (RP-LC). The RP-LC fractions were directly plated onto MALDI plates online. Tandem mass spectrometric data were acquired using 4800 Proteomics Analyzer-TOF/TOF (Applied Biosystems) linked to 4000 Series Explorer 3.0.

### Metabolomics

50 mg of liver sample was added to 150 μl of physiological saline for homogenate. Then 500 μl methanol were added, followed by 1 min of vortex mixing and 10 min of standing at -20 ℃ for protein precipitation. After centrifugation at 12000 g for 10 min at 4 ℃, 300 μl of the supernatant was transferred to an autosampler vial and blown to dryness with nitrogen. The residue was dissolved in 80 μl of methoxyamine (15 mg/ml) and the methoximation reaction was carried out for 90 min of shaking at 30 ℃, then 50 μl of BSTFA containing 1% TMCS was added for another 1 h of thimethylsilylation at 70℃. At last, a 1 μl of aliquot of the solution was injected into an Agilent 6890 GC system coupled with 5975B mass spectrometer (Agilent technologies, USA) for analysis.

Chromatographic separation was carried out on a capillary column (Agilent J&W DB-5ms Ultra Inert, 30 m * 0.25 mm * 0.25 μm) using programmed temperature showed in Table 1. Parameters in mass spectrometer were as follows: scan range, m/z 30–550; temperature of injection, interface and source, 280 ℃, 260 ℃ and 230 ℃.

**Table 1:** Programmed temperature of GC/MS.

Rate (℃/min)	Temperature (℃)	Hold time(min)
	80	2
5	93	0
8	109	0
5	185	2
10	255	1
10	290	7
PostRun	300	5

### Western blot

Hepatic proteins were extracted and quantified by BCA assay kit (Boster, Wuhan, China). The experiment was constructed as previously published method [13]. Briefly, equal amounts of proteins were electrophoresed andelectrotransfered. Antibodies specific for ALDH1B1 (LSBio, Seattle, USA), ALDH2 (Abcam, UK), ALDH7A1 (Proteintech, Chicago, USA) and GAPDH (CST, USA) were used. Anti-mouse IgG and anti-rabbit IgG (Yeasen, Shanghai, China) were used for detection of primary antibodies and internal control. Signals were imaged with chemiluminescent substrate kit (Thermo Scientific, Rockford, USA) and detected by gel imaging system (Tanon, Shanghai, China), and the ratio of interested proteins to GAPDH was analyzed by Tanon Gis software.

### Data analysis and statistics

Protein identification and quantification were performed with Maxquant 1.3.0.5. Proteins were considered up- or down-regulated if the fold change ≥ 1.2 or ≤ 0.83, respectively. Heatmap was carried out using SBC analysis system (SBC Shanghai Biotechnology Co., Ltd., Shanghai, China). Log-log plot was generated by GraphPad Prism 5.0 (San Diego, California, USA). Gene ontology (GO) and pathway analysis was performed using DAVID database (http://david.abcc.ncifcrf.gov/home.jsp).

Metabolite identification and quantification were carried out using Agilent MSD workstation. Data was normalized with the sum of all peaks, and then normalized data was imported to SIMCA-P 11 (Umetrics AB, Umea, Sweden) for principle component analysis (PCA), partial least squares-discriminant analysis (PLS-DA) and orthogonal partial least squares (OPLS). The permutation analysis was performed by the PLS-DA. The calculation of variable importance in the projection (VIP) was got by the OPLS. Metabolites were considered statistically significant if VIP > 1 and *P* value <0.05. Identification was performed by searching in NIST database, and pathway analysis was carried out in KEGG database (http://www.kegg.jp/).All data of pharmacological evaluation were expressed as mean ± SD and statistically analyzed using One-Way ANOVA test (SPSS 16.0, Chicago, IL, USA), and *P* value <0.05 was considered statistically significant.

## Results

### Gypenoside alleviated histological changes of liver fibrosis

**Fig 1:**
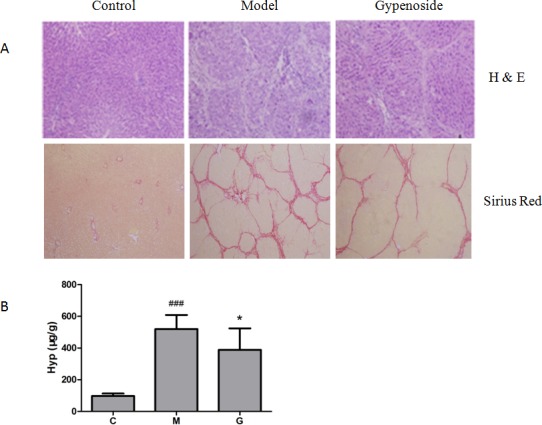
Effects of gypenoside on histological changes of liver fibrosis rats. **(A)** H&E staining (*200) and Sirius red staining (*100). **(B)** Hyp content. (C: control group; M: liver fibrosis model group; G: gypenoside group. Data are shown as mean ± SD. ### *P*<0.001 (vs. Control); * *P*<0.05 (vs. Model).)

Liver tissue sections were determined to evaluate the extent of hepatic fibrosis development by H&E stain and Sirius red stain. The results showed that no morphological abnormality and collagen staining were observed in control group. In model group, liver injury and fibrosis were obvious, including hepatocytic necrosis, continuous infiltration of neutrophils, and various collagen depositions. Compared to these histological changes in model group, there were prominentimprovements in gypenoside-treated group (Fig 1A). Furthermore, liver Hyp content was measured to evaluate the effect of gypenoside against fibrosis. Compared to control group, liver Hyp content was significantly increased in model group (*P*<0.001, Fig 1B), while it was statistically decreased by gypenoside (*P*<0.05). These results indicated that CCl_4_ exposure induced the formation of liver fibrosis in rats, and gypenoside put off the progression of liver fibrosis.

### Alteration of protein profiling in gypenoside-treated liver fibrosis rats

To determine the protein profiling in control group, model group and gypenoside-treated group respectively, we carried out proteomic analysis of liver tissue in rat using iTRAQ technology. After normalization of the data, a total of 2627 proteins were identified, of which 597 proteins were ≥ 1.2 or ≤ 0.83-fold change in gypenoside-treated group versus model group, including 301 up-regulated and 296 down-regulated ones.

**Fig 2:**
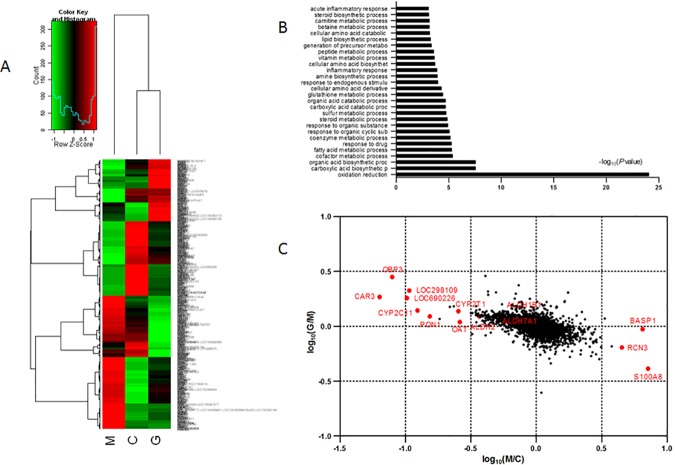
Proteomics analysis of gypenoside-treated liver fibrosis rats. **(A)** Heatmap of the significantly altered proteins in control, model and gypenoside group. Red represents an up-regulation, and green represents a down-regulation in protein expression. The proteins were clustered into four broad clusters. **(B)** Representative Gene Ontology (GO) biological processes (*P*<0.001) of differentially expressed proteins between gypenoside and model groups. **(C)** Log-log plot of the two conditions M/C and G/M based on protein expression ratios. Outstanding protein expression changes are indicated according to their gene names. (C: control group; M: liver fibrosis model group; G: gypenoside group.)

Cluster analysis in the expression of those significantly regulated 597 proteins is showed in Fig 2A, the table of gene symbols was provided as S1 Table, and cluster analysis in the expression of those all 2627 proteins is showed in S1 Fig. In Fig 2A, the heatmap was used to group the proteins into four main clusters in order to better explain the altered biological processes in their distribution across the proteomics data, also discriminate the groups into two clusters, that is the model group and the others. It suggested that considerable variability existed in proteome-wide expression between model group and control and gypenoside-treated groups.

To further elaborate biological significance of regulated proteins, an enrichment analysis was constructed to functionally annotate them into related biological processes of GO map. Fig 2B represents the significantly changed biological processes of *P* value < 0.001 between model group and gypenoside-treated group.“Oxidation reduction” emerged as the top network, whose–log_10_(*P* value) was 24, much higher than the others. Various biosynthetic and catabolic processes were found to be differentially regulated, including carboxylic acid, organic acid, amino acid and so on. Additional networks altered included inflammatory response, as well as a number of metabolic processes.

Strikingly regulated proteins among control group, model group and gypenoside-treated group are showed in Fig 2C. The proteins further away from origin represented those more strongly up- and down-regulated resulting from liver fibrosis-state or gypenoside-treatment. Proteins which located in the second quadrant represented down-regulated in model group versus control group and up-regulated in gypenoside-treated group versus model group, such as CAR3, which participated in nitrogen metabolism; PON1, related to steroid, sterol and cholesterol metabolic process, carboxylic acid and organic acid catabolic process, amino acid derivative metabolic process, etc. Proteins which located the fourth quadrant represented up-regulated in model group versus control group and down-regulated in gypenoside-treated group versus model group, such as RCN3, involved oxidation reduction; S100A8, related to inflammatory response, wounding response and defense response.

### Alteration of metabolite profiling in gypenoside-treated liver fibrosis rats

**Fig 3:**
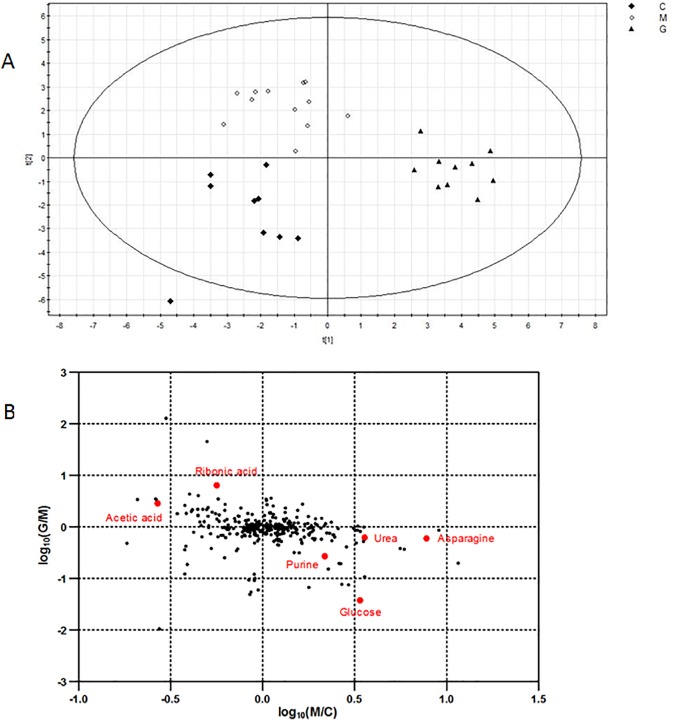
Metabolomics analysis of gypenoside-treated liver fibrosis rats. **(A)** Score plots of PCA analysis of control group, model group and gypenoside group. **(B)** Log-log plot of the two conditions M/C and G/M based on metabolite expression ratios. Outstanding metabolite expression changes are indicated according to their names, and other unlabeled outstanding metabolites were not identified. (C: control group; M: liver fibrosis model group; G: gypenoside group.)

To explore the metabolite profiling in control group, model group and gypenoside-treated group respectively, we performed metabolomic analysis of liver tissue in rats using GC/MS technology (Data shown in S2 Table). After normalization and statistical analysis of the data, a total of 350 metabolite peaks were detected, of which 17 significantly different metabolites were found and identified in gypenoside-treated group versus model group, including 9 up-regulated and 8 down-regulated ones (Table 2). Moreover, the metabolites of rat liver tissue in control group, model group and gypenoside-treated group were obviously discriminated to the three different groups by PCA (Fig 3A), implying that there were different metabolite profiling of rats liver tissues in the three groups. The validation plot of permutation analysis supported the validity of data (S2 Fig).

**Table 2:** Significantly altered metabolites and their pathways.

No	Compound	VIP	*P* value	G/M	M/C	Pathway
1	Acetic acid	1.5	0.001	↑	↓ **	Carbohydrate metabolism
2	Ribonic acid	1.3	0.028	↑	↓ *	Carbohydrate metabolism
3	Ribitol	1.0	0.044	↓	↑	Carbohydrate metabolism
4	Galactonic acid	1.3	0.018	↑	↑	Carbohydrate metabolism
5	L-Fucose	1.1	0.026	↓	↓	Carbohydrate metabolism
6	D-Glucose	2.0	<0.001	↓	↑ ***	Carbohydrate metabolism
7	D-Mannose	1.3	0.015	↑	↑ ***	Carbohydrate metabolism
8	Glycine	1.9	<0.001	↑	↑ ***	Amino acid metabolism
9	L-Alanine	1.9	<0.001	↑	↓ **	Amino acid metabolism
10	L-Serine	1.8	<0.001	↑	↑ **	Amino acid metabolism
11	L-Ornithine	1.7	<0.001	↓	↑ **	Amino acid metabolism
12	Putrescine	1.5	0.002	↑	↓ **	Amino acid metabolism
13	Valeric acid	1.3	0.005	↓	↑ **	Lipid metabolism
14	Glutaric acid	1.7	0.001	↑	↑	Lipid metabolism
15	Dodecanoic acid	1.2	0.017	↓	↓ ***	Lipid metabolism
16	Eicosenoic acid	1.4	0.003	↓	↑ ***	Lipid metabolism
17	Purine	1.5	0.001	↓	↑ **	Nucleotide metabolism
18	Phosphoric acid	1.9	<0.001	↑	↓ ***	Energy metabolism
19	Terephthalic acid	1.0	0.046	↓	↓ *	Xenobiotics biodegradation and metabolism

* *P*<0.05

** *P*<0.01

*** *P*<0.001

Similarly with proteomics data, log-log plot was constructed for significantly altered metabolites using metabolomics data (Fig 3B). Metabolites which were lower in model group than control group and higher in gypenoside-treated group than model group were found, such as acetic acid and ribonic acid. Metabolites which were higher in model group than control group and lower in gypenoside-treated group than model group were also showed, such as glucose, asparagine, urea and purine. Name, VIP and *P* value of statistically different metabolites in gypenoside-treated group compared to model group were listed in Table 2. The 17 significantly altered metabolites were invloved in kinds of pathways, such as carbohydrate metabolism, amino acid metabolism, lipid metabolism, nucleotide metabolism and so on. 13 of those 17 metabolites altered statistically in model group compared to control group, including 7 up-regulated and 6 down-regulated ones.

### Alterations of metabolic pathways by integrative analysis of proteomics and metabolomics data

According to integrative analysis of proteomics and metabolomics data, some key pathways were found in which both proteins and metabolites were significantly altered after gypenoside treatment, such as glycolysis or gluconeogenesis, fructose and mannose metabolism, glycine, serine and threonine metabolism, lysine degradation, arginine and proline metabolism, glutathione metabolism, and sulfur metabolism (Table 3).

**Table 3:** Significantly altered pathways with differentially expressed proteins and metabolites.

No	Term	Proteins	Metabolites
1	Glycolysis / Gluconeogenesis		
		ACSS2, ALDOA, ALDOB, ALDOC, ALDH1A3,**ALDH1B1**, **ALDH2**, ALDH3A2,**ALDH7A1**, ALDH9A1, BPGM,PKLR	D-Glucose, Acetate
2	Fructose and mannose metabolism		
		ALDOA, ALDOB, ALDOC, AKR1B1, KHK, SORD	L-Fucose, D-Mannose
3	Glycine, serine and threonine metabolism		
		AMT, BHMT, CHDH, DAO, PSPH, SDS	Glycine, L-Serine
4	Lysine degradation		
		ACAT1, **ALDH1B1**,**ALDH2**, ALDH3A2, **ALDH7A1**, ALDH9A1, BBOX1, HADH,TMLHE	Glycine, Glutarate
5	Arginine and proline metabolism		
		**ALDH1B1**, **ALDH2**, ALDH3A2, **ALDH7A1**, ALDH9A1, DAO, OCT, PRODH, PRODH2, PYCRL	L-Ornithine, Putrescine
6	Glutathione metabolism		
		GGCT, GGCTL1, GSTA2, GSTA3, GSTK1, GSTP1, GSTT1, LOC494499, MGST1, MGST2, TXNDC12	Glycine, L-Ornithine, Putrescine
7	Sulfur metabolism		
		BPNT1, SULT1E1, SULT2A2, SULT2AL1	Acetate, L-Serine

**Fig 4:**
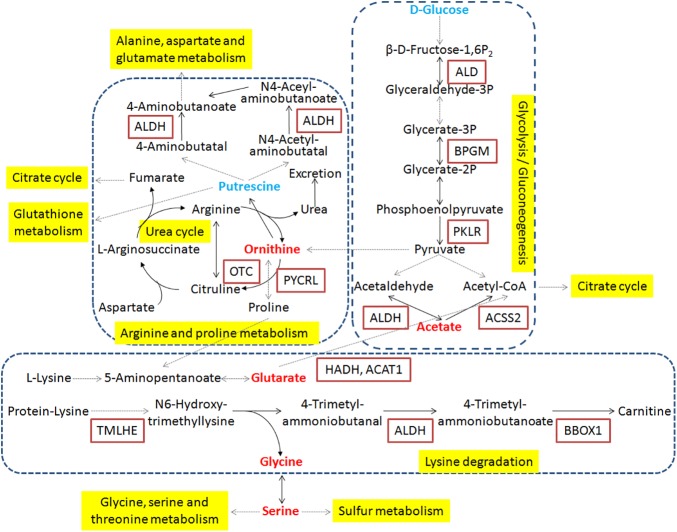
The networks of significantly altered metabolic pathways in response to gypenoside. Pathways are showed in yellow dashed areas. Proteins are showed in rectangles, and metabolites are showed in no rectangles. Red represents up-regulation in gypenoside group compared to model group, and blue represents down-regulation.

Notably, acetaldehyde dehydrogenases (ALDHs) were strikingly altered in three pathways, including glycolysis orgluconeogenesis, lysine degradation, and arginine and proline metabolism, implying that ALDH may play a crucial role in the improvement of liver fibrosis with gypenoside treatment. As shown in Fig 4, we picked those altered metabolic pathways for more detailed analysis of proteins and metabolites. Most proteins and metabolites in those pathways were up-regulated, and several were down-regulated, such as D-glucose and ornithine, implying that those molecules and metabolic pathways were associated with gypenoside treatment.

### Regulation of key proteins in altered metabolic pathways

**Fig 5:**
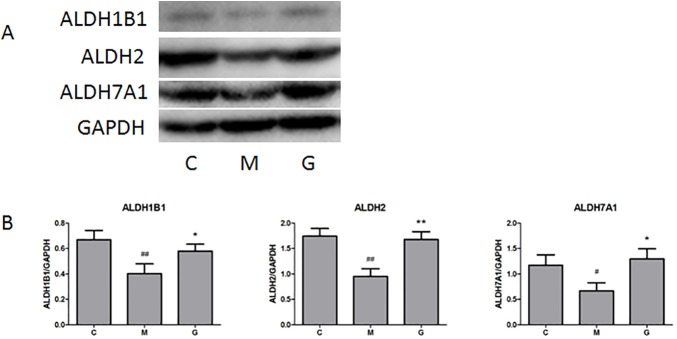
Effect of gypenoside on hepatic protein levels. Hepatic protein levels of ALDH1B1, ALDH2 and ALDH7A1 were examined in control, model and gypenoside groups by western blot. Hepatic GAPDH level was used as loading control. (C: control group; M: liver fibrosis model group; G: gypenoside group. Data are shown as mean ± SD. # *P*<0.05, ## *P*<0.01 (vs. Control); * *P*<0.05, ** *P*<0.01 (vs. Model).)

Considering the importance of ALDH, the levels of ALDH1B1, ALDH2 and ALDH7A1 expressions were analyzed by western blot. As Fig 5A and 5B shown, while the levels of ALDH1B1, ALDH2 and ALDH7A1 expressions were significantly reduced in model group, gypenoside recovered them, which were consistent with proteomics data shown in Fig 2C.

## Discussion

Active compounds derived from plant extracts are utilized to treat more and more clinical diseases [14]. Identifying efficacy and mechanism of those compounds from nature is particularly important and meaningful. Gypenoside is a kind of saponin derived from *Gynostemmapentaphyllum* (Thunb.) Makino., a Chinese herbal medicine. Previous studies demonstrated that gypenoside have hepatoprotective activity. For example, gypenoside could reduce collagen content and inhibit liver fibrosis[3], protect against ischemia/reperfusion-induced hepatic injury[4], and also prevent liver fatty degeneration and ameliorate fatty liver disease[15]. In this study, histological examination and Hyp content assay showed that hepatocellular damage and collagen deposition were distinguishable in CCl_4_-treated group, while the fibrosis state was apparently inhibited in gypenoside-treated group (Fig 1). Thus, our results further indicated that gypenoside could inhibit CCl_4_-induced liver fibrosis.

Liver fibrosis results from chronic damage to the liver in conjunction with the excessive accumulation of extracellular matrix (ECM) proteins[16]. The fibrotic liver contains approximately 6 times ECM more than normal, which is a result of both increased synthesis and decreased degradation [17]. Our previous study explored the mechanisms of CCl_4_-induced liver fibrosis with combined transcriptomic and proteomic analysis [18]. On the other hand, when liver fibrosis occurs, a host of metabolic pathways will alter in hepatocytes, and related proteins and metabolites will be also involved hepatic disorder. Glycolysis is a universal metabolic pathway for providing energy. In the early phase of liver injury, energy metabolism is maintained by increasing energy production from glycolysis because of the weak ability of oxidative phosphorylation, while at the terminal stage, hepatocytes are unable to sustain the increased demand for high levels of energy production from glycolysis [19]. Furthermore, many researchers found that perturbation of amino acid metabolism appeared in liver disease patients or liver injury rats, such as branched-chain amino acids, aromatic amino acids, and so on [20–22].

In the present study, we investigated the profiles of proteins and metabolites in CCl_4_-induced liver fibrosis rats and gypenoside-treated rats, and found some significantly altered molecules and pathways, involving glycolysis and some amino acids metabolism (Fig 4). After treated with gypenoside, D-glucose was decreased, acetate was increased, and most enzymes were increased in glycolysis pathway, thus glycolysis was up-regulated. Just like other metabolic pathways, glycolysis was regulated following the sensitivity of key enzymes to changing conditions in the cells [23]. Redirecting energy metabolism towards glycolysis has been certified to minimize oxidative damage and suppress apoptosis [24]. Previous studies suggested that some therapies blocked the progression of liver fibrosis involving up-regulation of glycolysis or related proteins in glycolysis pathway [25, 26], which was also consistent with our study.

Considering ALDHs were strongly altered in multiple pathways, it implied that ALDH may play a key role in gypenoside inhibiting liver fibrosis. ALDHs are a family of enzymes to modulate some cell functions, including survival, proliferation, differentiation, cellular response to oxidative stress, and so on. They show a rather broad substrate specificity and many of them can oxidize some highly reactive aromatic and aliphatic aldehydes which act as a pivot in mediating various physiological, pathological and pharmacological processes [27]. Lipid peroxidation (LPO) induces the formation of more than 200 highly reactive and toxic aldehyde species, such as malondialdehyde (MDA). ALDH is known as “aldehyde scavenger” and can protect against various environmental stressors [28]. In this study, we chose to validate ALDH1B1, ALDH2 and ALDH7A1 highly expressed in liver, whose major substrates included acetaldehyde etc. which occurred in the altered pathways of this study. They were found up-regulated in gypenoside-treated rats, perhaps, in order to protect against the damage of aldehydes. In our previous study, FuZheng HuaYu Decoction (FZHY) was found decreased MDA content [29], and gypenoside was one component of FZHY. It implied that gypenoside maybe attenuate the damage of MDA and LPO by up-regulating ALDHs. Furthermore, it also suggested that the alteration of ALDH1B1, ALDH2 and ALDH7A1 levels may be potential biomarkers for the curative effect evaluation of gypenoside against liver fibrosis.

In conclusion, gypenoside improved histopathological changes of hepatic dysfunction in CCl_4_-induced liver fibrosis rats, and the pharmacological effect of inhibiting fibrosis may be involved in the alteration of glycolysis metabolism and the protection against the damage of aldehydes and LPO by up-regulating ALDH.

Previous study showed that olive oil had a protective effect against CCl_4_ with regards to fibrosis [30]. However, the influence of olive oil has not been discussed in this study. Whether the beneficial effect of olive oil involve in the regulation of fatty acids levels, or other aspects, which need to be explored in further study.

## Supporting information

S1 Table Gene symbols of significantly regulated 597 proteins between gypenoside group and model group. (XLSX)

S2 Table Metabolomic data of liver tissue in rats using GC/MS technology. (XLSX)

S1 Fig Cluster analysis in the expression of those all 2627 proteins. (PNG)

S2 Fig Validation of the PLS-DA model by 50 permutations of data. (DOCX)
