# Supplementary figures and images for: Metabolomic mechanisms of gypenoside against liver fibrosis in rats: An integrative analysis of proteomics and metabolomics data
Source: PLoS One. 2017 Mar 14;12(3):e0173598. doi: 10.1371/journal.pone.0173598 (PMC5349658; doi:10.1371/journal.pone.0173598)

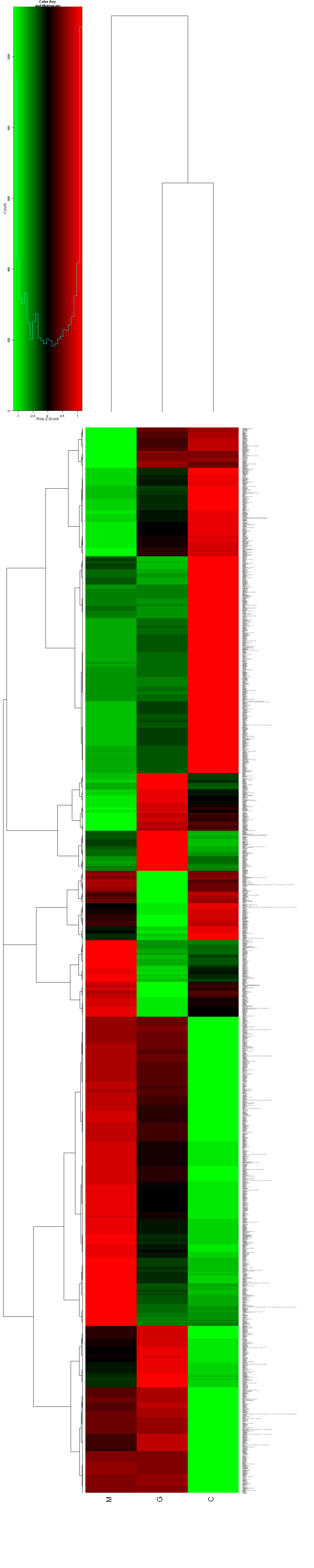

Supplement: S1 Fig — (PNG) [file pone.0173598.s003.png]

Supplemental Figure S2


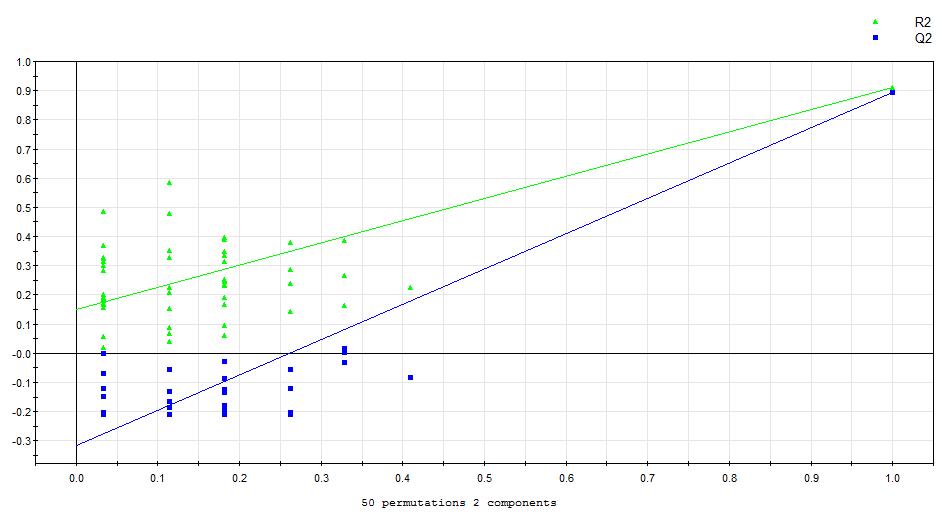


Figure S2. Validation of the PLS-DA model by 50 permutations of data.

Supplement: S2 Fig — (DOCX) [file pone.0173598.s004.docx]
